# Supplementary material for: Systemic Oxidative Balance Reflects the Liver Disease Progression Status for Primary Biliary Cholangitis (Pbc): The Narcissus Fountain
Source: Antioxidants (Basel). 2024 Mar 23;13(4):387. doi: 10.3390/antiox13040387 (PMC11047334; doi:10.3390/antiox13040387)
Supplement: Supplementary file 1 [file antioxidants-13-00387-s001.zip › antioxidants-2905783-supplementary.pdf]

Supplementary File S1.

EVALUATION OF LIFESTYLE HABITS

Physical exercise assessment:

Medical validated questionnaire and relative items assessing physical exercise practicing in enrolled patients.

Questions/Items	Answer	
Are you doing or have you ever done (in the last 2 years) sport in a continuative and regular way?	YES	NO
Have you changed your daily physical activity in the last 3 months?	NO	YES
If yes, has it enhanced or worsened?	Enhanced	Worsened
How many **hours per week** do you usually spend for physical exercise?	More than 150 minutes/week	Less than 150 minutes/week

Each patient was considered on **active** physical exercise if he/she has done sports in the last 2 years, this practice has not worsened in the last 3 months by spending at least 150 minutes per week in physical activity.

Supplementary Table S1. Nutritional assessment in study population groups relative to the period of 3-month controlled regimen diet

Variables		Healthy (n:10)	PBC (n:41)	MASLD (n:40)	CHB (n:52)	CHC (n:50)	*p*-value of the comparison between the CLDs etiologies
Physical activity (hours/week in the last 3 months) (mean ± SD)		6.3 ± 1.5	6.1 ± 1.3	6.1 ± 1.2	5.8 ± 1.3	5.9 ± 1.4	PBC vs Healthy: n.s.<br>PBC vs MASLD: n.s.<br>PBC vs CHB: n.s.<br>PBC vs CHC: n.s.
Patients (%) on "active physical exercise"		75.1	78.2	71.9	74.5	73.8	PBC vs Healthy: n.s.<br>PBC vs MASLD: n.s.<br>PBC vs CHB: n.s.<br>PBC vs CHC: n.s.
Daily intake (Kilocalories / day)		2305 ± 817.4	2307 ± 411.5	2307 ± 408.7	2305 ± 410.2	2306 ± 521.3	PBC vs Healthy: n.s.<br>PBC vs MASLD: n.s.<br>PBC vs CHB: n.s.<br>PBC vs CHC: n.s.
Carbohydrates (Kilocalories)		1072± 241.4	1073 ± 233.1	1075 ± 230.3	1071 ± 243.2	1078 ± 417.6	PBC vs Healthy: n.s.<br>PBC vs MASLD: n.s.<br>PBC vs CHB: n.s.<br>PBC vs CHC: n.s.
Lipids (Kilocalories)		579.3 ± 242.1	572.3 ± 131.2	580.9 ± 131.4	577.2± 238.3	574 ± 248.9	PBC vs Healthy: n.s.<br>PBC vs MASLD: n.s.<br>PBC vs CHB: n.s.<br>PBC vs CHC: n.s.
Proteins (Kilocalories)		658.4 ± 202.3	662.2 ± 196.8	658.7 ± 199.6	659.3 ± 201.6	666.7 ± 162.4	PBC vs Healthy: n.s.<br>PBC vs MASLD: n.s.<br>PBC vs CHB: n.s.<br>PBC vs CHC: n.s.

PBC: Primary Biliary Cholangitis; CHB: Chronic HBV infection; CHC: Chronic HCV infection; MASLD: Metabolic dysfunction associated steatotic liver disease; SD: standard deviation. In the case of non-normal or normal distribution, the Kruskal-Wallis test or ANOVA test with post-hoc Tukey analysis, respectively, were performed to compare the continuous variables among different CLDs etiologies. Statistically significant differences (p<0.05) are reported in bold.
